# Effect of closing material on hearing rehabilitation in stapedectomy and stapedotomy: A finite element analysis

**DOI:** 10.3389/fnins.2023.1064890

**Published:** 2023-02-14

**Authors:** Jongwoo Lim, Woonhoe Goo, Dae Woong Kang, Seung Ha Oh, Namkeun Kim

**Affiliations:** ^1^Department of Mechanical Engineering, Korea Advanced Institute of Science and Technology (KAIST), Daejeon, Republic of Korea; ^2^Department of Otorhinolaryngology, Seoul National University Hospital, Seoul, Republic of Korea; ^3^Department of Mechanical Engineering, Sogang University, Seoul, Republic of Korea

**Keywords:** stapedectomy, stapedotomy, hearing loss, finite element model, closing material

## Abstract

Stapedotomy or stapedectomy operations are often performed to treat otosclerosis. During the operation, the space created by bone removal is usually filled with a closing material such as fat or fascia. In this study, the effect of the Young’s modulus of the closing material on the hearing level was investigated through the 3D finite element model of a human head including auditory periphery. The Young’s moduli of the closing material used to implement stapedotomy and stapedectomy conditions in the model were varied from 1 kPa to 24 MPa. The results showed that the hearing level improved when the closing material was more compliant after stapedotomy. Therefore, when the stapedotomy was performed using fat whose Young’s modulus is lowest among the potential closing materials, the hearing level recovered the best among all simulated cases. On the other hand, in stapedectomy, the Young’s modulus did not have the linear relationship between the hearing level and the compliance of the closing material. Hence, the Young’s modulus causing the best hearing rehabilitation in stapedectomy was found not at the end of the investigated range of Young’s modulus but somewhere in the middle of the given range.

## 1. Introduction

Otosclerosis is a disease in which the stapes annular ligament (SAL) is stiffened, resulting in conductive hearing loss (CHL) (Chole and McKenna, 2001; Menger and Tange, 2003; Ealy and Smith, 2011). Owing to the stiffened SAL, sound energy cannot be transmitted into the cochlea through the ossicles of the middle ear. To resolve the malfunctioning stapes, two types of operations are usually performed: stapedotomy and stapedectomy. To allow the sound energy to be transmitted into the cochlea, a hole is drilled in the stapes footplate, and a prosthesis attached on the incus is inserted into the hole during the stapedotomy (Jean, 1985; Perkins, 1980). In the operation, the gap between the hole and prosthesis is usually filled by fascia tissue or fat graft (Quaranta et al., 2005; Lin and Selesnick, 2016). On the other hand, in the stapedectomy, the stapes footplate is either partially or completely removed, corresponding to a partial or total stapedectomy, respectively, and the empty space is filled with fat or fascia (Sürala et al., 1969; Persson et al., 2009; Sakamoto et al., 2015). However, there have been conflicting views on determining better remedies in terms of the patients’ hearing recovery between the stapedectomy and stapedotomy. There are numerous studies showing that the stapedotomy enables better hearing rehabilitation than the stapedectomy (Bailey et al., 1981; Pedersen and Elbrønd, 1983; Moon and Hahn, 1984; House et al., 2002; Colletti and Fiorino, 2016; Somers et al., 2016). For example, according to House et al., the stapedotomy showed statistically better initial and late post-operative 4-kHz air-conduction thresholds than the stapedectomy (House et al., 2002). On the other hand, only few references state that the stapedectomy allows better hearing rehabilitation (Persson et al., 2009); according to them, hearing levels of patients who underwent total stapedectomy recovered by about 7 dB more after 1 year of follow up and by 6 dB more after 3 years of follow up in a pure tone average (PTA) than those of patients who underwent the stapedotomy. A few other studies have noted that there were no substantial differences between both surgical techniques and that both have been reported to yield similar clinical outcomes (Cremers et al., 1991; Sedwick et al., 1997; Quaranta et al., 2005; Vasama et al., 2006; Scarpa et al., 2022). Based on these observations, it has been generally accepted that the stapedotomy is safer and more effective for helping patients with otosclerosis than the stapedectomy (Bailey et al., 1981; Pedersen and Elbrønd, 1983; Moon and Hahn, 1984; House et al., 2002; Colletti and Fiorino, 2016; Somers et al., 2016). Despite the numerous studies on the clinical outcomes of operations, the reason for better hearing rehabilitation from the stapedotomy is unclear. Specifically, the effects of the diameter of the prosthesis (Shabana et al., 1999; Marchese et al., 2007) and perforation size in the stapes footplate on hearing rehabilitation (Kos et al., 2016) have been investigated, but there are very few studies on the closing material for the hole or removed stapes footplate. In fact, there is debate on whether a larger piston diameter can have more advantageous effects on hearing (Marchese et al., 2007). Simplistically, it seems mechanically plausible that a larger movable area on the stapes footplate can allow better hearing rehabilitation. Furthermore, this logic could be expanded to opinions that the stapedectomy can allow better hearing rehabilitation than the stapedotomy. To explain these results in a stricter sense, the Young’s modulus of the closing material, such as fat or fascia, should be considered. Since the cochlear input impedance is defined as the pressure in the scala vestibuli near the stapes footplate over volume velocity of oval window, the stiffness and the damping of the closing materials are significant factors to determine the cochlear input impedance at low and mid frequencies, respectively.

In this study, we aim to investigate the effects of the closing material on the hearing levels after stapedotomy or stapedectomy operations. Using a three-dimensional (3D) finite-element (FE) model, the basilar membrane (BM) velocity was calculated by varying the Young’s modulus of the closing material to evaluate the recovered hearing levels after the operations.

## 2. Materials and methods

The used abbreviations in this study are summarized in Table 1.

**TABLE 1:** Summary of abbreviations for the present study.

Abbreviations	Definition
AC	Air conduction
BAHA	Bone-anchored hearing aid
BC	Bone conduction
BF	Best frequency
BM	Basilar membrane
CHL	Conductive hearing loss
FE	Finite element
HL	Hearing level
PTA	Pure tone average
SAL	Stapes annular ligament
SPL	Sound pressure level
TM	Tympanic membrane

### 2.1. Finite-element model

**FIGURE 1:**
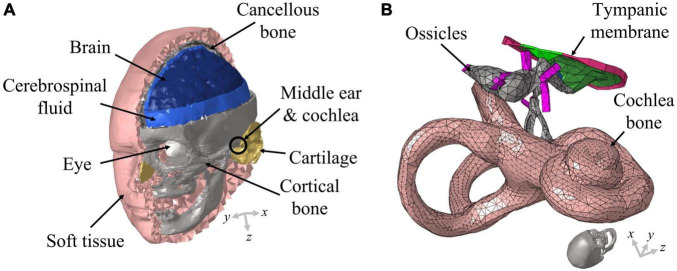
Schematic of the full-head model used in this study. **(A)** The head model consists of the brain, cerebrospinal fluid, eyes, soft tissue, cortical bone, cartilage, and cancellous bone; **(B)** the auditory periphery model consists of the middle ear and cochlea. The auditory periphery model is located in an appropriate position within the head model. The ends of the ligaments and the outer cochlear bone are connected with the skull in the head model.

The present study was conducted with a previously developed 3D FE full-head model (Kim et al., 2014; Chang et al., 2016; Lim et al., 2021). Although a detailed description of the 3D FE full-head model can be found in literature (Lim et al., 2021), a brief explanation is provided here for readers’ convenience. The 3D FE full-head model contains an auditory periphery; the geometry of the human head was obtained by segmentation based on cryosectional images of a cadaver head (Chang et al., 2016) and that of the auditory periphery was acquired by segmentation based on micro computed tomography images (Kim et al., 2014). The FE head model was composed of about 258,000 solid and 144,000 fluid tetrahedral elements. On the other hand, the auditory periphery FE model comprised approximately 104,000 solid and 507,000 fluid tetrahedral elements. Specifically, the BM in the auditory periphery was composed of about 18,000 solid pentahedral elements whose top and bottom surfaces were triangles. The geometry of the 3D FE full-head model is shown in Figure 1A and that of the auditory periphery is shown in Figure 1B.

**FIGURE 2:**
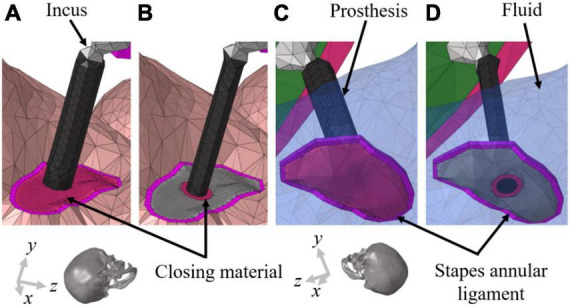
Geometrical illustrations of the stapedectomy and stapedotomy in the right ear of finite-element (FE) models. **(A)** To implement stapedectomy, the entire stapes was removed, and the closing material was filled in the space from which the stapes footplate was removed and a prosthesis (φ = 0.6 mm) connecting the end of the incus to the center of the closing material was added. **(B)** The stapedotomy involved placing a hole (φ = 0.6 mm) in the stapes footplate to insert the prosthesis (φ = 0.4 mm); the stapes footplate and prothesis are connected by the closing material. Near the stapes footplate, the cochlear fluid is in contact with **(C)** only the closing material in stapedectomy, whereas the fluid in the stapedotomy is in contact with **(D)** the end of the prosthesis, stapes footplate, and closing material. The transparent blue elements in **(C,D)** represent cochlear fluid.

Although the head model was validated several times in the previous studies, the closing material elements to represent the stapedectomy were newly generated with lower Young’s modulus. Therefore, the volume velocity of the oval window replaced by the closing materials was calculated to confirm the convergence with decreasing the mesh size of the elements as 0.2, 0.1, 0.05, and 0.02 mm (see Figures 2A, B). Since mesh-converged results for the volume velocity were achieved at a mesh size of 0.05 mm, was determined as the mesh size to represent the closing materials in stapedectomy conditions.

### 2.2. Material properties

Most material properties of the full-head model were identical to those in the previous study, except for the cortical and cancellous bones (diploë) in Figure 1A (Kim et al., 2014; Chang et al., 2016; Lim et al., 2021). In the previous studies, the Young’s modulus values of the cortical and cancellous bones were somewhat lower in the range of reference, *i.e.*, 8 and 0.4 GPa, respectively, so that the model would have consistent promontory accelerance and best frequency (BF) map with the reference (Lim et al., 2021). However, the Young’s moduli for the skull were tuned to 20 GPa and 200 MPa to satisfy the Carhart’s notch in this study (Carhart, 1950). In other words, the cortical bone having a Young’s modulus of 8 GPa cannot show hearing loss at around 1–2 kHz for bone conduction (BC), which is clinically observed in patients with otosclerosis. Based on the changes in the Young’s moduli for the skull, the promontory accelerance (*i.e.*, acceleration divided by input force), BF map, and BC hearing loss from the otosclerosis condition were recalculated for model validation, as shown in the “Results” section. Furthermore, the detailed material properties of head and auditory periphery models are summarized in Tables 2, 3, respectively (Kim et al., 2014; Chang et al., 2016; Lim et al., 2020, 2021).

**TABLE 2:** Properties of 3D finite-element head model.

Component	Young’s modulus, *E* [MPa]	Density, ρ [kg/m^3^]	Loss factor, η (constant)
Brain	35 × 10^–3^ (Kleiven and Hardy, 2002; Luo et al., 2012; Sahoo et al., 2013)	1 × 10^3^ (Luo et al., 2012)	0.3
Eye	1,500 [m/s; sound speed] (Levin et al., 1981)	1 × 10^3^ (Levin et al., 1981)	0.1
Cortical bone	20 × 10^3^ (Turner et al., 1999)	1.8 × 10^3^ (Peterson and Dechow, 2003)	0.3
Diploë	2 × 10^2^ (Jaasma et al., 2002)	1 × 10^3^ (Kleiven and Hardy, 2002; Luo et al., 2012; Sahoo et al., 2013)	0.75 (at 1 kHz)
Cerebrospinal fluid	1,500 [m/s; sound speed] (Levin et al., 1981)	1 × 10^3^ (Levin et al., 1981)	0.1
Cartilage	7.5 (Grellmann et al., 2006)	1 × 10^3^ (Maroudas et al., 1969)	0.3
Soft tissue	0.7 (Kim et al., 2010)	0.9 × 10^3^ (Farvid et al., 2005)	0.03
Screw	2 × 10^5^ (Tjellström et al., 1981)	7.85 × 10^3^ (Tjellström et al., 1981)	–

Mechanical properties (Young’s modulus, density, and loss factor) of the FE head model components. Specifically, fluids are characterized by the sound speed instead of Young’s modulus. The loss factor, η, is the ratio of the imaginary to real parts of the complex Young’s modulus. Note that the loss factor of the diploë increases linearly with frequency.

**TABLE 3:** Properties of 3D finite-element auditory periphery model.

Component	Young’s modulus, *E* [MPa]	Density, ρ [kg/m^3^]	Loss factor, η (constant)
Incus ossicle	1.41 × 10^4^ (Gan et al., 2004)	2.15 × 10^3^ (Sim et al., 2007)	0.01
Malleus ossicle	1.41 × 10^4^ (Gan et al., 2004)	2.39 × 10^3^ (Sim et al., 2007)	0.01
Stapes ossicle	1.41 × 10^4^ (Gan et al., 2004)	2.20 × 10^3^ (Gan et al., 2004)	0.01
Malleus/incus joint	7 (Homma et al., 2010)	1.2 × 10^3^ (Gan et al., 2004)	0.15
Incus/stapes joint	0.44 (Gan et al., 2004)	1.2 × 10^3^ (Gan et al., 2004)	0.15
Tensor tympani	5 (Homma et al., 2010)	1.2 × 10^3^ (Homma et al., 2010)	0.3
Anterior ligament	5 (Homma et al., 2010)	1.2 × 10^3^ (Homma et al., 2010)	0.3
Lateral ligament	10 (Homma et al., 2010)	1.2 × 10^3^ (Homma et al., 2010)	0.3
Superior ligament	2 (Homma et al., 2010)	1.2 × 10^3^ (Homma et al., 2010)	0.3
Stapes tendon	3.8 × 10^3^ (Gan et al., 2004)	1.2 × 10^3^ (Homma et al., 2010)	0.15
Incus ligament	4.8 (Gan et al., 2004)	1.2 × 10^3^ (Homma et al., 2010)	0.15
Tympanic membrane, pars tensa	2 × 10^1^ (Homma et al., 2010)	1.2 × 10^3^ (Koike et al., 2002)	0.7
Tympanic membrane, pars flaccida	7 (Homma et al., 2010)	1.2 × 10^3^ (Koike et al., 2002)	0.15
Tympanic annulus	0.6 (Koike et al., 2002)	1.2 × 10^3^ (Homma et al., 2010)	0.5
Stapes annular ligament	0.7 (Kim et al., 2014)	1.2 × 10^3^ (Homma et al., 2010)	0.3
Round window	0.05 (Kim et al., 2014)	1.2 × 10^3^ (Kim et al., 2014)	0.8
Cochlear bone	20 × 10^3^ (Turner et al., 1999)	1.8 × 10^3^ (Peterson and Dechow, 2003)	0.3
Cochlear fluid	1,500 [m/s; sound speed] (Levin et al., 1981)	1 × 10^3^ (Levin et al., 1981)	0.1
Basilar membrane	6.5–5.5 (longitudinally from base to apex) 0.2–1 × 10^3^ (transversely from base to apex) (Kim et al., 2014)	1 × 10^3^ (Kim et al., 2014)	0.3
Prosthesis	150 × 10^3^ (Salvinelli et al., 2009)	21 × 10^3^ (Salvinelli et al., 2009)	0.01
Closing materials	0.001, 1, 24 (Comley and Fleck, 2010a,b; Trindade et al., 2012; Zwirner et al., 2020)	1.2 × 10^3^ (Ward and Lieber, 2005)	0.3

Mechanical properties (Young’s modulus, density, and loss factor) of the auditory periphery model components. Specifically, the fluid is characterized by sound speed instead of Young’s modulus. The loss factor, η, is the ratio of the imaginary to real parts of the complex Young’s modulus.

### 2.3. Otosclerosis and post-surgical simulations

The Young’s modulus of the normal SAL has been considered to be around 0.7 MPa (Kim et al., 2014). However, to implement the otosclerosis condition in this simulation, the Young’s modulus of the SAL was increased to that of the cortical bone, meaning that otosclerosis indicates a fully ossified SAL in this study. In general, the SAL of otosclerosis patients is neither completely ossified especially in earlier stages nor uniformly ossified along the perimeter. However, in the current study, it was assumed that the SAL is completely and uniformly ossified to focus on the effect of the closing material on the hearing threshold. This can also reduce the complexity in analysis caused by various contributing factors. To study the effects of the closing materials used in stapes surgeries on CHL, the geometries of the stapes and stapes footplate were modified corresponding to each surgical method. In the simulation, the stapedectomy was implemented by removal of the entire stapes. The closing material was then inserted into the space from which the stapes footplate was removed. Furthermore, a prosthesis (φ = 0.6 mm) connecting the end of the incus to the center of the closing material was added. To implement the stapedotomy conditions in the FE model, the stapes head, anterior crus, and posterior crus were removed while retaining the stapes footplate. A hole (φ = 0.6 mm) was then placed at the center of the footplate to insert the prosthesis (φ = 0.4 mm) connecting the end of the incus to the hole. The closing material was then used to fill the small gap between the hole and prosthesis completely. The stapedectomy and stapedotomy performed in the simulations are described in Figures 2A, B, respectively. It should be noted that the cochlear fluid in the scala vestibuli is in contact with the closing material only near the stapes footplate in the stapedectomy, whereas the cochlear fluid near the stapes footplate in the stapedotomy is in contact with the stapes footplate and prosthesis as well as the closing material. Figures 2C, D show the detailed contact conditions of the cochlear fluid near the stapes footplate. In general, since the surgical sites of the stapedectomy and stapedotomy are closed or filled with fascia or fat, the properties of the closing materials in the simulations were determined as those of fascia or fat. According to previous studies, the Young’s modulus of the fascia was reported to be in the range of 1–24 MPa (Trindade et al., 2012; Zwirner et al., 2020) whereas that of fat was reported to be about 1 kPa (Comley and Fleck, 2010a,b). In Figures 2B, D, the center of the prosthesis and footplate could be not perfectly aligned according to the calculation method to find the center of the asymmetric-oval shape. However, it was confirmed that a slight change in the position of the hole did not significantly affect the hearing level. Therefore, the simulations were conducted with shown geometry in Figure 2.

### 2.4. Air- and bone-conduction stimulation

**FIGURE 3:**
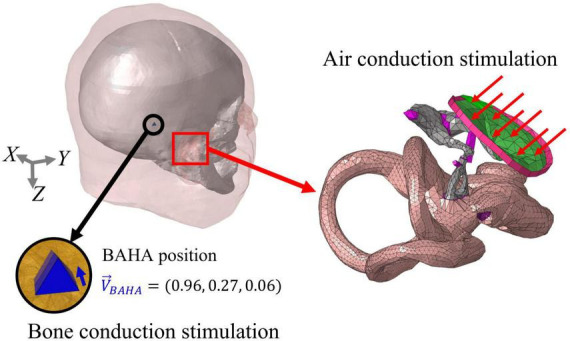
Stimulation methods of air-conducted (AC) and bone conduction (BC) hearing. In AC stimulation, a distributed pressure is applied on the TM, with the outer bony shell of the cochlea, ends of the ligaments, and edge of the tympanic annulus being fixed. On the other hand, the BC input was stimulated on the screw elements at the BAHA position. The force was applied in the longitudinal direction of the screw and represented as a unit vector.

In this study, it is assumed that the velocity of the BM (V_*BM*_) is related to the hearing threshold in both air-conducted (AC) and bone-conducted (BC) hearing. The AC stimulation was implemented by assigning a uniformly distributed dynamic unit pressure on the surface of the tympanic membrane (TM). Boundaries of the head and auditory periphery components, such as the ends of the ligaments and tendons, edge of the tympanic annulus, and outer bony shell of the cochlea, were fixed. On the other hand, the BC excitations were implemented by assigning a sinusoidal force on the screw component in the typical position for a bone-anchored hearing aid (BAHA). The screw component was inserted perpendicular to the skull surface at the BAHA position. The direction of the sinusoidal force was determined as the direction in which the screw component was inserted. Figure 3 shows the stimulus methods for AC and BC hearing as well as the directions of the corresponding sinusoidal forces. All the simulations were performed using the commercial software ACTRAN (MSC Software, Newport Beach, CA, USA) in the frequency domain from 0.1 to 10 kHz in 0.1 kHz increments.

To obtain the BF map of the current FE cochlear model, the BM velocities were calculated at about 180 positions (every about 0.2 mm from the base to the apex) along the BM length at each simulated frequency. Based on these calculated velocities, the specific position showing the maximum velocity among the 180 positions corresponding to an input frequency was defined as the ‘BF position’. In addition, since the BF position is the same between the normal and the specific condition except when the input frequency is different, the hearing loss (or gain) was calculated by the difference in BM velocities at the BF position according to the input frequency between the normal and the specific condition (e.g., otosclerosis).

### 2.5. Clinical data for stapedotomy operation

For the model validation, we compared the simulation results with clinical data obtained from stapedotomy operations. The Institutional Review Board of Seoul National University College of Medicine/Seoul National University Hospital approved this study (IRB No. 2209-120-1360) and waived the need for informed consent owing to the retrospective design and use of anonymous clinical data for analyses. A total of six patients (10 ears) who were diagnosed with otosclerosis between January 2020 and May 2020 and underwent stapedotomy were included in the study. The mean age of in total six patients who were involved in clinical analysis was 44.3 ± 17.9 years. A platinum piston wire prosthesis (Medtronic Xomed, Jacksonville, FL; modeled with 150 GPa Young’s modulus in the current FE model) was utilized and moved over the stapedotomy opening. The piston wire prosthesis was advanced into the vestibule and crimped over the incus. The prepared soft tissue was placed around the gap between the hole and prosthesis. The soft tissues were not distinguished as fat or fascia in the operations. The results of the pre-operative and post-operative PTAs at 1 year after surgery were collected and analyzed. It should be noted that masking was performed to prevent cross hearing in the measurement when the hearing threshold difference between both ears in a patient exceeded the interaural attenuation.

## 3. Results

### 3.1. Validation

**FIGURE 4:**
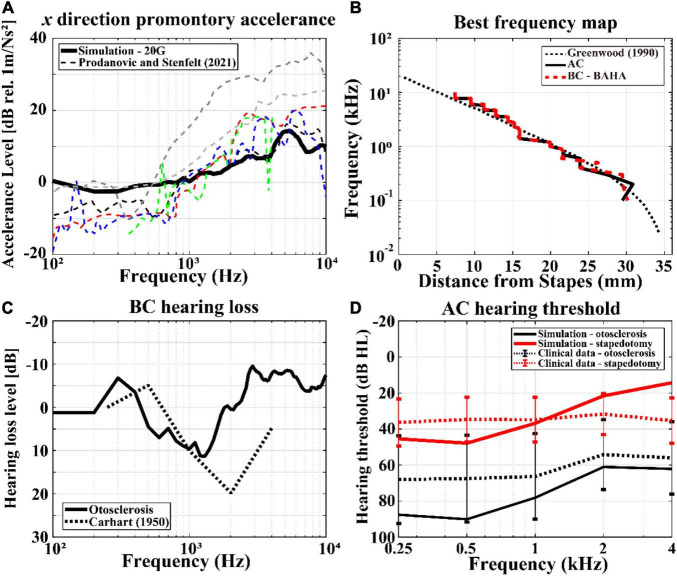
Validations of the current finite-element (FE) models. The simulation results are represented by solid lines, whereas those of the previous studies or clinical data are shown as dashed lines: **(A)** promontory accelerance, **(B)** BF maps according to AC and BC stimulations, **(C)** BC hearing losses including Carhart’s notch, and **(D)** hearing thresholds in AC stimulation with otosclerosis (black lines) and stapedotomy (red lines) conditions. In **(D)**, the error bars represent the 95% confidence interval of the measurements’ mean.

Although the full-head model was validated in a previous study, revalidation of the model was needed owing to the changes in the Young’s moduli of the skull in the present study. Therefore, the promontory accelerance, BF map, and BC hearing loss in otosclerosis were recalculated, as shown in Figure 4. There are many previous studies on measurement of the promontory accelerance (Stenfelt and Goode, 2005; Eeg-Olofsson et al., 2008, 2011; Håkansson et al., 2008; Hakansson et al., 2010; Rigato et al., 2018; Dobrev et al., 2019); Prodanovic and Stenfelt summarized the results of promontory accelerance (Prodanovic and Stenfelt, 2021). Therefore, we compared the calculated promontory accelerance with the experimental results reported by Prodanovic and Stenfelt in Figure 4A, where the calculated accelerance is indicated by the black solid line and the previous experimental results are shown by dashed lines of various colors. The simulation results of the present work were within the range of experimental values although the results showed a tendency to be in the upper range of the reference values below 1 kHz and in the lower range above 1 kHz. Similar to the promontory accelerance, the BF map of the present work is reasonably consistent with those of previous studies regardless of the AC or BC condition, as shown in Figure 4B. As mentioned before, the Carhart’s notch has been clinically reported for patients with otosclerosis (Carhart, 1950). However, the Carhart’s notch cannot be observed when the Young’s modulus of the cortical bone is 8 GPa. Thus, the Young’s modulus of the cortical bone was modified to 20 GPa to obtain the Carhart’s notch that was consistent with clinical data, as shown in Figure 4C. Approximately 10 dB of BC hearing loss was calculated in the frequency range of 1–2 kHz from the otosclerosis condition, which is similar to clinical data.

The hearing threshold level can be defined as the pressure level on the TM when the human perceives a sound. At this time, the pressure level represented by sound pressure level (SPL) on the TM can be converted into 0 dB hearing level (HL). For example, according to ISO 8253-1, a 13.5 dB SPL results in human perception of sound at 0.5 kHz. Thus, 13.5 dB SPL is considered as 0 dB HL at 0.5 kHz (ISO, 2022a). The HLs of pre-operative and post-operative conditions in the simulations are compared with those of clinical data, as shown in Figure 4D. In the figure, “otosclerosis” and “stapedotomy” represent pre-operative and post-operative conditions, respectively. To calculate the AC HL, the reference equivalent threshold SPL in ISO 389-1 was used to obtain the minimum BM velocity at the hearing threshold (ISO, 2022b). In other words, the pressure in the ISO 389-1 describing the pressure on the TM at the hearing threshold for a normal ear was applied on the TM in the current model. Then, the HL pressures for otosclerosis or stapedotomy conditions were calculated by satisfying the BM velocities obtained at the hearing thresholds in the normal ear, and the calculated HL pressures are converted to dB HL according to ISO 8253-1. In Figure 4D, the simulation results are shown by solid lines, whereas the clinical data are described by dotted lines. The black and gray lines indicate otosclerosis (*i.e.*, pre-operative) and stapedotomy (*i.e.*, post-operative) conditions, respectively. The error bars in Figure 4D represent the standard deviations calculated from 10 data sets. It should be noted that audiometry was conducted at specific frequencies of 250, 500, 1,000, 2,000, and 4,000 Hz, for which the clinical data exist.

The AC HLs in post-operative simulations were within the standard deviation of the clinical data along the input frequency changes. The AC HLs for the pre-operative simulations were within the standard deviation of the clinical data at high frequencies above 2 kHz. However, there were 20–40 dB differences between the simulation results and clinical data at low frequencies below 2 kHz. This difference is further explored in the “Discussion” section.

### 3.2. Hearing loss corresponding to post-operative condition

**FIGURE 5:**
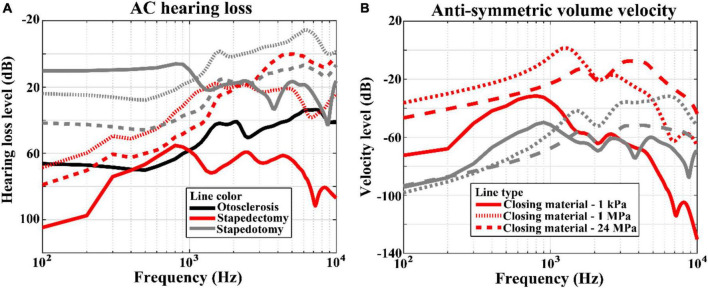
**(A)** Hearing losses and **(B)** anti-symmetric volume velocities between the oval window and round window for the AC simulation results. The line styles represent variations in the Young’s moduli of the closing materials. The solid, dotted, and dashed lines correspond to 1 kPa, 1 MPa, and 24 MPa, respectively. On the other hand, the line color indicates the simulation condition; the black, red, and gray lines describe otosclerosis, stapedectomy, and stapedotomy conditions, respectively.

The hearing losses in the pre-operative and post-operative conditions were calculated by subtracting the BM velocities for each condition from those for the normal condition. Since the BF position is the same regardless of the operation condition unless the input frequency is different, the position where the BM velocities are calculated was the BF position corresponding to the input frequency. The hearing losses of otosclerosis (*i.e.*, pre-operative) are represented by black solid lines in Figure 5, whereas those of stapedectomy and stapedotomy (*i.e.*, post-operative) conditions are shown with red and gray lines, respectively. Specifically, the post-operative cases are separated into three types whose Young’s moduli of the closing materials are 1 kPa, 1 MPa, and 24 MPa, which are represented by solid, dotted, and dashed lines, respectively.

Figure 5A shows the hearing loss corresponding to the AC input. The hearing losses in stapedotomy were proportional to the Young’s moduli of the closing materials. In other words, as the Young’s modulus of the closing material increased, the hearing loss increased. On the other hand, the AC hearing losses in stapedectomy did not show a proportional relationship with the Young’s moduli of the closing materials. When the Young’s modulus increased from 1 kPa to 24 MPa, the largest hearing loss occurred at the 1 kPa closing material and the smallest hearing loss occurred at the 1 MPa closing material. Figure 5B indicates the anti-symmetric volume velocities between the oval window and round window for each of the cases. In this figure, the proportional or non-proportional relationships between the anti-symmetric volume velocities and Young’s moduli of the closing materials are repeatedly observed, as also seen in Figure 5A.

## 4. Discussion

### 4.1. Hearing level in AC

In the process of validation, although most results from the promontory accelerance, BF map, and hearing loss in the BC condition for the Carhart’s notch were reasonably consistent with those of previous studies, the AC HL of the otosclerosis simulation [black solid line in Figure 4D] showed large differences from the average values of the clinical data [black dotted line in Figure 4D]. More specifically, the hearing level in the simulation was about 20 dB lower than that of the clinical data at frequencies below 1 kHz. However, the stiffness of the SAL has not been measured for otosclerosis patients in the clinical data. Although the hearing loss level is large enough to be diagnosed with otosclerosis, the SAL may not be perfectly ossified. On the other hand, the stiffened SAL was implemented by a perfectly ossified case using the cortical bone properties in the simulations. Therefore, the discrepancy at low frequencies can be explained by the difference in the Young’s modulus of the SAL. The larger hearing loss from the simulation is thus acceptable unless the patient’s SAL is completely ossified. Furthermore, activation of different hearing pathways can be another reason for the difference in hearing thresholds. While a patient can hear a sound through both AC and BC pathways, the FE model is stimulated by the AC mechanism only (*i.e.*, dynamic pressure on TM). This difference can cause the higher hearing threshold in the model than that in a patient. In addition, the simulation and clinical data show about 10–20 dB difference in hearing level at 4 kHz. However, the large error bars indicate that there are large variations in the patients’ responses. As mentioned above, various statuses of patient SALs may cause these large errors. For further study, it is necessary to measure the SAL stiffness in otosclerosis patients before stapedotomy or partial stapedectomy surgery, as different occlusive materials may be used depending on the type of surgery. Since the cochlear input impedance of a post-operative patient can be determined by a combination of the SAL and the closing material, the optimized closing material in accordance with operation type for the patient can be effectively determined through the SAL stiffness. The relevance between the closing material and operation type is explained below.

### 4.2. Hearing loss corresponding to post-operative condition

**FIGURE 6:**
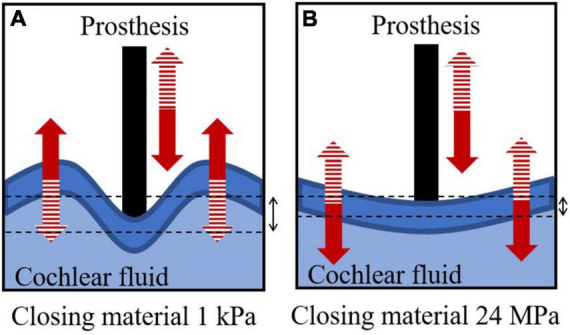
Descriptions of cochlear fluid, prosthesis, and closing material in stapedectomy when the Young’s modulus of the closing material is **(A)** 1 kPa and **(B)** 24 MPa. The solid and dashed patterns in the arrows represent the same phase of deformation.

The AC hearing loss in stapedotomy is proportional to the Young’s modulus of the closing material, while there is no proportional relationship between the Young’s modulus of the closing material and hearing loss in the stapedectomy simulation. The non-proportional relationship in stapedectomy can be explained by the deformed shape of the closing material replacing the stapes footplate. In the stapedotomy, a large portion of the stapes footplate remains despite the perforation. Therefore, the remaining stapes footplate shows in-phase motions due to its high stiffness. However, in the stapedectomy, the stapes footplate was thoroughly replaced by the closing material; thus, when the Young’s modulus of the closing material was sufficiently compliant, such as 1 kPa, although the area of the prosthesis tip moved into the cochlear fluid, the closing material surface bulged into the cochlear fluid, as described in Figure 6A (see the left panel of Supplementary Video). This causes less anti-symmetric volume velocity [red solid lines in Figure 5B], resulting in less rehabilitation from hearing loss [red-solid lines in Figure 5A] (Kim et al., 2011). On the other hand, when the Young’s modulus was high, *i.e.*, 24 MPa, the high stiffness caused less movement but greater in-phase motions of the closing material, as described in Figure 6B (see also the right panel of Supplementary Video). It should be noted that the closing material with high stiffness in the stapedectomy shows different movements from the stapes footplate under normal conditions because the SAL is assumed to be fully ossified in the post- as well as pre-operative conditions. In summary, in the stapedectomy operation, the closing material should be appropriately compliant or stiff so as to (1) prevent different phase shapes of the closing material with movement of the prosthesis tip and (2) permit the closing material to move smoothly. Therefore, the 1 MPa closing material showed the best rehabilitation in the current study. Based on this observation we suggest a possibility to change the prevailing thought regarding operation for otosclerosis patients. Since the area enclosed by the closing material in the stapedectomy is larger than that of the stapedotomy, the stapedectomy is generally considered to improve AC hearing more than the stapedotomy. Although the stapedectomy may lead to better hearing rehabilitation, the stapedectomy is considered to have higher risk such as perilymphatic fistulae or leakage, need for revision, and damage to the facial nerve over the oval window. Hence, the stapedotomy is the preferred operation for otosclerosis patients these days. However, according to the simulation results, the stapedotomy shows better hearing rehabilitation than the stapedectomy when using appropriate closing materials. For example, if fat is used for the closing material in the stapedotomy, then the hearing ability can be recovered up to the level achieved by a stapedectomy with fascia as the closing material.

Although the current study using the FE model clarified the effect of the closing material on the hearing level, the following limitations should be considered for better understanding of stapedectomy and stapedotomy. Firstly, the current FE model has a different boundary condition than a clinical case for the connection between the prosthesis and the incus. While the prosthesis is attached to the lenticular process in the current model, a prothesis is crimped to the long process (even to other locations) in real patients. Although the effect of the closing material stiffness on the hearing level could not be affected by the connection condition, the attaching condition can directly affect the hearing level. Therefore, variance of the connection condition can be a future study topic for better understanding of stapedectomy and stapedotomy. Secondly, after stapedectomy or stapedotomy operation, the state of the interface between the closing material and the bone around the closing material, or between the closing material and the prosthesis can change over time. Therefore, in the next investigation, the effect of the interface change over time should be considered for better understanding of hearing rehabilitation. In short, since the FE model inherently contains simplification, it cannot fully reflect the biological situation. Therefore, care is needed in the analysis of the results.

## 5. Conclusion

In this study, the simulations for otosclerosis and post-operative conditions were conducted using the 3D FE full-head model. The post-operative condition for stapedectomy was implemented by replacing the stapes footplate with compliant closing materials and connecting a prosthesis between the center of the closing material and the incus. On the other hand, the condition for stapedotomy was represented by placing a hole at the center of the stapes footplate and inserting a prosthesis connected with the incus into the hole. The gap between the prosthesis and the hole was filled with closing materials. The V_BM_ was used to calculate hearing loss, and the effects of the Young’s moduli of the closing materials on hearing losses in the AC case were investigated. According to the results, the level of hearing loss was proportional to the Young’s modulus of the closing material in the stapedotomy. On the other hand, there exists an optimized Young’s modulus of the closing material in the stapedectomy that allows the best hearing rehabilitation. Based on the results, more compliant closing materials can be used to achieve better hearing rehabilitation in the stapedotomy, whereas an appropriate Young’s modulus of the closing material is able to improve hearing ability in the stapedectomy. Hence, fat is better as a closing material than fascia in the stapedotomy, whereas the fascia with 1 MPa Young’s modulus is better than the other types of fascia (with Young’s modulus of 24 MPa) or fat in the stapedectomy.

## Data availability statement

The original contributions presented in this study are included in this article/Supplementary material, further inquiries can be directed to the corresponding authors.

## Ethics statement

The studies involving human participants were reviewed and approved by Seoul National University College of Medicine and Seoul National University Hospital. Written informed consent for participation was not required for this study in accordance with the national legislation and the institutional requirements.

## Author contributions

JL: simulations, formal analysis, and writing – original draft. WG: methodology, data curation, and validation. DK: methodology, data curation, and writing – original draft. SO: conceptualization, supervision, and writing – review and editing. NK: conceptualization, supervision, writing – review and editing, and project administration. All authors contributed to the article and approved the submitted version.
